# Problem drinking and exceeding guidelines for 'sensible' alcohol consumption in Scottish men: associations with life course socioeconomic disadvantage in a population-based cohort study

**DOI:** 10.1186/1471-2458-8-302

**Published:** 2008-09-01

**Authors:** G David Batty, Heather Lewars, Carol Emslie, Michaela Benzeval, Kate Hunt

**Affiliations:** 1 MRC Social & Public Health Sciences Unit, University of Glasgow, Glasgow, UK

## Abstract

**Background:**

With surveys suggesting that exceeding guidelines for 'sensible' alcohol intake is commonplace, the health and social impact of modifying intake on a population level is potentially considerable. If public health interventions are to be successfully implemented, it is first important to identify correlates of such behaviours, including socioeconomic disadvantage. This was the aim of the present study.

**Methods:**

Population-representative cohort study of 576 men from the West of Scotland. Data on life course socioeconomic position were collected in 1988 (at around 55 years of age). Alcohol consumption patterns (detailed seven day recall) and problem drinking (CAGE questionnaire) were ascertained in 1990/2 (at around 59 years of age). A relative index of inequality was computed to explore the comparative strength of different indicators of social circumstances from different periods of the life course.

**Results:**

Socioeconomic adversity in both early life and in adulthood was related to an increased risk of exceeding the weekly and daily alcohol guidelines, with adult indicators of socioeconomic position revealing the strongest associations. Of these, material indicators of socioeconomic deprivation in adulthood – car ownership, housing tenure – were marginally more strongly related to heavy alcohol intake and problem drinking than education, income and occupational social class. A substantial proportion of the influence of early life deprivation on alcohol intake was mediated via adult socioeconomic position. Similar results were apparent when problem drinking was the outcome of interest.

**Conclusion:**

In men in this cohort, exposure to disadvantaged social circumstances across the lifecourse, but particularly in adulthood, is associated with detrimental patterns of alcohol consumption and problem drinking in late middle age.

## Background

The study of the health consequences of heavy alcohol consumption has a long research tradition: the first investigations of risk were undertaken in the early 19^th ^century by life assurance companies who calibrated their premiums according to the alcohol consumption of policy holders [1]. Subsequently, more detailed studies have reported "U"- or "J"-shaped associations between alcohol intake and both all-cause and coronary heart disease mortality [2]. That is, while abstainers and heavy consumers experience elevated risk, moderate drinkers do not.

Based on these data, and in keeping with other health behaviours such as dietary intake [3] and physical activity [4], guidance on appropriate alcohol intake has been widely disseminated. Present recommendations for 'sensible' weekly consumption (up to 21 units in men and 14 units in women) were first advanced by the *Royal College of Physicians *[5] over two decades ago and subsequently endorsed by other health agencies leading to official adoption by the UK government [6] and those of many other nations. There is growing empirical evidence that drinking above and beyond these weekly limits has a detrimental impact on health and that, more specifically, exceeding a recently proposed daily benchmark of 4 units (men) and 3 units (women) carries "....an increasingly significant risk of illness and death from a number of conditions....." [7]. A negative impact on non-health outcomes, such as family breakdown and financial hardship, has also been suggested [8], and has received some support in the few population-based studies conducted [9].

With over half of the adult population of England reporting alcohol consumption in excess of the new daily guidelines [10], the health and social impact of modifying intake on a population level is potentially considerable. However, if public health interventions are to be successfully implemented it is crucial to identify the predictors of such behaviours. A recent review proposed a series of risk factors for heavy drinking, including a lack of awareness about the potential harms, use of other substances, drinking as a coping strategy, and poor family relations [11]. Some of these risk indices correlate with socioeconomic position, which, with few exceptions [12], reveals strong relationships with binge drinking, although the magnitude and direction of this gradient tends to vary by gender [13] and particularly country [14-16].

While understanding of the socioeconomic variations in binge drinking is growing and informative, there remain important gaps. First, there are relatively few general population-based studies: many investigators utilise occupational cohorts [17-20] which have narrow socioeconomic variation which may lead to associations with alcohol intake being underestimated. Second, there has been a recent revitalisation of interest in life course predictors of chronic disease such as ischaemic heart disease, cancer, and psychiatric illness. While the role of behavioural processes underlying these relationships – particularly smoking – has also been well examined [21,22], with the exception of two studies [23,24], little is known about life course predictors of heavy drinking, in particular exceeding the afore-described guidelines for 'sensible' alcohol consumption. Evidence for a strong influence of pre-adult social factors on later heavy drinking would point to the need for intervention earlier in life than is currently the case. In a study of Finnish middle-aged men, unfavourable levels of a range of life course markers of socioeconomic position, particularly those from adult life, were predictive of binge drinking [23]. Similar results were apparent in a Scottish cohort which used self-reported hangovers as a proxy for heavy alcohol use [24]. However, both studies were characterised by crude indicators of alcohol intake and complex data interpretation, such that a direct comparison between early and later life influences on binge drinking was problematic given the differing coding structure for each predictor variable.

Using data from the West of Scotland Twenty-07 study, we are able to address several of these shortcomings: participants completed a detailed seven day recall of alcohol intake; we utilised the relative index of inequality which facilitates a direct comparison of the relative strength of the association of different markers of socioeconomic disadvantage at different stages of the life course with the risk of exceeding existing guidelines for sensible weekly and daily alcohol intake; and a wider range of socioeconomic indices was collected than has previously been the case. Additionally, for the first time, we report on life course socioeconomic inequalities in drinking problems, as ascertained by validated questionnaire, which in itself is associated with elevated mortality risk [25].

## Methods

Study participants are from the *West of Scotland Twenty-07 Study*, a population-sampled cohort designed to investigate the influence of social factors on health. Ethics committee approval for the Twenty-07 study was granted by the General Practice Subcommittee of the Greater Glasgow Health Board Area Medical Committee, and the West of Scotland General Practice Ethical Committee.

The design and sampling have been described in detail elsewhere [26,27]. In brief, the Twenty-07 study comprises three cohorts recruited at around age 15, 35, and 55 years in 1987/8. Our analyses are based on the oldest age group who, in wave one, took part in two interviews in the study participant's home, one enquiring about social circumstances, and another with a nurse about health-related factors (N [proportion of target sample]: 851 [88%] in women; 700 [88%] in men). In wave 2 (1990/2; age around 59 years), attempts were made to re-interview these men and women about their alcohol drinking habits and any related problems. Again, response was high (681 [83%] in women; 578 [87%] in men).

### Assessment of life course socioeconomic position

Early socioeconomic circumstances were based on four indices. Paternal occupational social class was coded into one of six categories according to the Registrar General's schema [28]. Family structure was denoted by the presence of both biological parents at age 15 years. Respondents also reported their number of siblings, and age at leaving school (range: 12–19 years). Assessment of adult socioeconomic circumstances is based on seven indices. Occupational social class was coded as above. To categorise their employment status, study participants identified themselves as: retired, disabled/invalid, caring for the home/housewife, in education, unemployed (no paid work) or employed/worker/self employed. Income was based on total household earnings after tax, including any benefits; respondents were asked to specify an actual amount in pounds sterling per week, month or year, or, if they were unwilling to do so, to identify an appropriate income band on a preprinted card. Housing tenure was categorised as privately owned or other (council, privately rented [unfurnished], privately rented [furnished], tied to job). Household crowding was calculated by dividing the number of people in the household by the number of rooms; respondents were then assigned to a quartile of the distribution, with the highest quartile representing most overcrowding. Study participants also indicated whether or not they owned a car or van. Finally, for marital status, subjects responded to a series of enquiries which allowed them to be categorised into one of three groups: married, no longer married (separated, widowed, divorced), or never married (single).

### Assessment of alcohol consumption and problem drinking

Study participants provided a recall of their alcohol consumption over each of the seven days preceding the interview, reporting separately for five categories of alcohol type: beer (including lager and cider), wine, fortified wine, spirits, and 'other' (e.g., 'alcopops'). Responses were expressed in units which represent 8 grams of pure alcohol, equivalent to half a pint of ordinary beer, lager, or cider, a small glass of wine, or a single measure of spirits. For weekly alcohol intake, data were totalled and respondents were dichotomised on the basis of whether or not they exceeded the recommendations for sensible weekly intake (21 units for men, 14 units for women) [5]. For daily intake, the number of days in the preceding seven on which a study participant exceeded 4 units (men) and 3 units (women) [7] was computed; respondents who had exceeded these guidelines on at least one day in the previous week were classed as 'heavy daily' drinkers. Both alcohol outcomes were categorised into whether or not they surpassed these limits (referred to here as "heavy weekly" or "heavy daily" drinkers).

All participants, with the exception of those who indicated they were lifelong non-drinkers, were asked to complete the CAGE questionnaire [29,30]. CAGE is an acronym based on the four questions that comprise the inventory: Have you ever felt you ought to *cut down *on drinking? Have people *annoyed *you by criticizing your drinking? Have you ever felt bad or *guilty *about your drinking? Have you ever had a drink first thing in the morning (*eye-opener*) to steady your hands? These items were used to create a simple drinking problem scale, with each positive response given a score of one; a higher score indicates the presence of an alcohol problem. A total CAGE score of 2 or more is considered clinically significant and this was used in the present analyses. While the CAGE does not provide standard Diagnostic and Statistical Manual diagnosis of alcohol dependence, a positive response on two or more questions indicates a high likelihood of the presence of problematic drinking [30].

### Statistical analyses

Based on these definitions of alcohol intake and problem drinking, preliminary analyses indicated there were too few women who could be classified as cases in order to facilitate analyses; we therefore focused on data from men only. We examined the relation of both early and later life socioeconomic position with these drinking outcomes using logistic regression modelling. Initially, our analyses were unadjusted, producing bivariate odds ratios. To explore linearity – an assumption inherent in the uses of the relative index of inequality (RII; see below) – we added a quadratic term to the model for each of the socioeconomic exposures variables when examining their relation with the three alcohol outcomes. As it was only possible to test for linearity for those predictor variables with three or more categories, we did not run the analyses for the dichotomously coded family structure, employment status, housing tenure and car ownership. The six remaining variables and three alcohol outcomes therefore resulted in 18 exposure-outcome permutations. Of these, in only one (housing crowding) did the quadratic term attain statistical significance at conventional levels. As this exceptional result is likely to be a chance finding, the linearity assumption can be regarded as not having been violated. Next, as we have done before [31], we calculated a RII to quantify the association of early and adult life exposures with drinking outcomes. Using the RII facilitates a comparison of effect estimates across a diverse range of indices of socio-economic position. Markers of socioeconomic position were recoded where necessary so that high values reflected disadvantage. The RII was then derived by ranking the participants on each of the socioeconomic measures. For the discrete measures, and in the case of ties for continuous measures, we assigned the mean rank. We then divided these rank scores by the sample size to yield a value between 0 and 1. For the purposes of interpretation, the RII should be regarded as the relative risk of exceeding the stated guidelines or problem drinking in the most disadvantaged group relative to the most advantaged. Its interpretation is the same as a relative risk ratio. Again, logistic regression was used to calculate a RII (odds ratio). The RII is known to elevate effect estimates, especially in variables with only two categories. However, because we used the RII solely as a comparison of the relation of the alcohol outcomes with socioeconomic variables which had different coding structures, our interest did not lie in the absolute size of these relationships.

We then calculated a lifetime composite score for socioeconomic adversity. To do so, we dichotomised all the explanatory variables (0, 1) so that experience of disadvantage on any measure contributed a single point to the score. Explanatory variables were dichotomised at the following demarcation points (for the categories implicitly classified as '0', see Additional file 1): father's and own social class (1 = partly skilled manual, unskilled manual); family structure (1 = circumstance other than having two biological parents in the family at age 15 years); education (1 = left school age 12–14 years); employment status (1 = all other categories but employed [i.e., retired, disabled/ill, caring for the home, in education, unemployed]); income (1 = lowest quartile); housing tenure (1 = all other categories except privately owned house [i.e., council, rent, job-related, other]); household crowding (1 = most overcrowded quartile); car ownership (1 = no); and marital status (1 = single).

Three indices were then created (early life socioeconomic position, range: 0–3; adult socioeconomic position, range: 0–7; life course socioeconomic position, range: 0–10). Again, a higher score indicated greater adversity. Each index was then assigned a RII using the procedure described above. Throughout all these analyses, the analytical sample varies slightly (range: 521–576) depending on missing data for the socioeconomic predictor of interest.

## Results

Of men with complete data on alcohol consumption (N = 576) and a response to enquiries about problem drinking (N = 578), 20.8% (N = 120) exceeded weekly and 44.6% (N = 258) daily guidelines for sensible consumption; 14.9% (N = 86) were categorised as having alcohol-related drinking problems. There was some overlap of cases according to each of the three alcohol outcomes. Thus, based on men with complete data on all alcohol outcomes (N = 576), of those who reported exceeding 'sensible' weekly intake of 21 units, 45% of these indicated that they consumed 4 units on one or more days per week. For the 'problem' drinking men in this cohort, 52% exceeded the weekly limit, while a predictably higher proportion (78%) surpassed the daily guidelines.

In Additional file 1 we present the relation of early life indicators of socioeconomic position with the three alcohol outcomes. There was a suggestion that study participants whose fathers were in more manual social classes reported an increased prevalence of heavy weekly drinking with an incremental effect seen across the occupational categories (p[trend] = 0.057). However, parental occupational social class was unrelated to either heavy daily or problem drinking at conventional levels of statistical significance. Conversely, both heavy daily alcohol consumption and problem drinking – but not heavy weekly intake – were positively related to number of siblings. While the confidence intervals for these effects contained unity, there was again some evidence of dose-response relations across the socioeconomic categories, particularly for problem drinking (p[trend] = 0.043). Education was related to all three alcohol outcomes, such that leaving school at 14 years of age or earlier conferred an elevated risk. Family structure was not related to any of the drinking outcomes in these analyses.

Additional file 2 shows the association between adult indices of socioeconomic position and the alcohol outcomes. Lower occupational social class in adulthood was related to an increased prevalence of heavy weekly (p[trend] = 0.045) and heavy daily alcohol intake (p[trend] = 0.003), and also to problem drinking (p[trend] = 0.004). Similarly, income revealed an inverse association with the alcohol outcomes. Men not living in privately owned accommodation, and men who reported not owning a car, were also more likely to surpass both weekly and daily drinking guidelines and to be categorised as having drinking problems. Although no substantial or consistent relationships were seen with employment status or marital status, there was a suggestion that single men and those not in employment exhibited less favourable drinking patterns than married and employed men, respectively. The relationship between household crowding and alcohol consumption was inconsistent.

Next, in order to compare the relative strengths of each index of socioeconomic position with the alcohol outcomes, we computed a RII. Additional file 3 presents early and later life socioeconomic predictors of heavy weekly drinking in ascending order of magnitude. Comparing disadvantaged men with advantaged, the strongest early life risk factor for heavy weekly drinking was a younger age at leaving school, while the weakest was family structure. In adult life, the strongest predictors were car ownership, housing tenure and current occupational social class; while the weakest were housing crowding and marital status. Confidence intervals were wide in some of these analyses. Similar patterns of association were apparent when heavy daily intake and problem drinking were the outcomes of interest.

Finally, again using the RII, we computed accumulative indices of socioeconomic adversity for early life, adult life and the life course and related these to each of the three alcohol outcomes (Additional file 4). Both early and adult life socioeconomic deprivation were associated with an increased risk of exceeding weekly and daily guidelines for sensible alcohol intake and for problem drinking. Total adult disadvantage was also related to each of the three alcohol outcomes. In comparison to childhood markers of deprivation, the gradients for adult deprivation were markedly stronger for heavy daily intake and problem drinking, and similar for heavy weekly intake. In order to examine if the early life index of disadvantage exerted an influence on the alcohol outcomes that was mediated via adult disadvantage, we controlled for the latter and noted a marked attenuation for each of the point estimates. Unsurprisingly, life course socioeconomic adversity was more strongly related to the alcohol outcomes than each of its components.

## Discussion

The aim of this study was to examine the association of socioeconomic disadvantage across the life course with the risk of exceeding existing guidelines for 'sensible' alcohol consumption and problem drinking. As indicated, only two studies [23,24] have, to our knowledge, examined the link between life course indicators of socioeconomic position and adult reports of heavy drinking that offer a lower level of detail than our own. A number of our results accord with these. First, we found that adult deprivation was more strongly related to our alcohol outcomes than early life deprivation [23,24]; second, material socioeconomic indicators in adulthood (car ownership, housing tenure) generally revealed a steeper gradient with heavy alcohol intake and problem drinking than other factors, such as education, income and occupational social class [24]; third, a substantial proportion of the influence of early life deprivation on alcohol intake was mediated via adult socioeconomic position [23]. We were also able, for the first time to our knowledge, to explore the link between life course social circumstances and a measure of self-reported alcohol problems which, like heavy drinking, is also associated with elevated mortality [25]. The pattern of association in these analyses was similar for our two indicators of alcohol intake.

### Study strengths and limitations

The present study has a number of strengths. First, the social class distribution of the study sample was very similar to a comparable group of the local population drawn from the UK's 1991 census samples of anonymised records, suggesting that our results are generalisable to the UK population [32]. Second, for an epidemiological investigation, the data on alcohol intake were unusually detailed. Third, participation in the surveys was high (≥85%), so minimising concerns about selection bias. Fourth, we were able to examine the link between alcohol outcomes and a wider range of socioeconomic markers than has previously been possible. Finally, our use of the relative index of inequality allowed us to compare the relative strength of different socioeconomic measures across different periods of the life course.

This study is not of course without its limitations. First, like most large scale studies, we relied on self-reported alcohol intake (crucial given the nature of our research question). However, agreement between self-report and biochemical markers of alcohol intake is sufficiently high for use in population-based studies [33]. Second, for data on early life socioeconomic circumstances, we relied on distant recall by middle-aged adults. In a systematic review, there was a suggestion that studies with prospectively collected data on childhood socioeconomic position tended to reveal somewhat stronger inverse associations with later mortality than studies with retrospective data [34]. However, over a similar period to that in the present study, adult recall of parental occupational social class shows moderate agreement with data collected (and archived) in early life [35]. Third, reverse causality is a plausible explanation for some of the relationships between socioeconomic position-alcohol intake/problems reported here. For instance, high alcohol intake and its attendant problems could lead to unemployment, reduced income, and loss of car ownership. This issue could be addressed by using alcohol outcomes based on incidence rather than prevalence, but we do not know when the study members became 'cases'. The problem of reverse causality is unlikely to be germane to the relation between alcohol consumption/problems and early life socioeconomic position when intake is likely to have been non-existent for most, if not all, study members. Finally, owing to a limited number of heavy/problem drinking women in the present study, we were unable to offer insights into the aetiological role, if any, of life course socioeconomic adversity in this group. Given the suggestion that other health-related outcomes, such as self-perceived health, reveal gender-specific relations with socioeconomic position in childhood and adulthood in analyses [36], it may be unwise to extrapolate these results to women. This therefore remains an understudied group.

### Public health context

Our results suggest that a range of socioeconomic indices are associated with heavy drinking and related alcohol problems in late middle age. Taking education as one of the more modifiable of these, the cognitive and socio-cultural characteristics of people with higher educational levels, for example the ability to obtain and synthesise health promotional materials, may be important. Educational experiences may also have a role in determining one's peers during sensitive periods across life course (late adolescence and early adulthood) when health behaviours, including pattern of alcohol intake, tend to be adopted [37]. Efforts to increase educational achievement are likely to be most profitable by targeting younger people. As has been highlighted [38], an adverse socioeconomic trajectory does not make decisions regarding more favourable health behaviours impossible, however, it may be associated with its own social constructions of which behaviours are perceived as being linked with optimal health or, as qualitative research of smoking has suggested, a means of coping with unfavourable social circumstances [39].

## Conclusion

Exposure to disadvantaged circumstances throughout the lifecourse, but particularly in adulthood, is associated with detrimental patterns of alcohol consumption and problem drinking in men in late middle age. Further research is needed to establish whether similar associations are seen in younger people and women, particularly given the increasing prevalence of alcohol consumption in these groups.

### What this paper adds

• This is one of the first studies to examine life course socioeconomic position as a predictor of problem drinking and heavy alcohol intake.

• Exposure to more disadvantaged circumstances throughout the life course, but particularly in adulthood, is associated with detrimental patterns of alcohol consumption and problem drinking in men in late middle age

### Policy implications

• Although a range of socioeconomic indices were linked with later detrimental alcohol intake and problem drinking, education may represent one of the most modifiable.

• Educational experiences may have a role in determining one's peers during sensitive periods across the life course (late adolescence and early adulthood) when health behaviours, including patterns of alcohol intake, tend to be adopted.

## Competing interests

The authors declare that they have no competing interests.

## Authors' contributions

The idea for the present manuscript was generated during discussions amongst the co-authors. GDB wrote the first draft of the manuscript based on data analyses conducted by HL. All authors commented extensively on subsequent revisions, and have read and approved the final manuscript.

## Pre-publication history

The pre-publication history for this paper can be accessed here:



## Supplementary Material

Additional file 1 Table 1. Odds ratios (95% CI) for the association of indices of early life socioeconomic position with heavy weekly, heavy daily and problem drinking in men. Click here for file

Additional file 2 Table 2. Odds ratios (95% CI) for the association of indices of adult socioeconomic position with heavy weekly, heavy daily and problem drinking in men. Click here for file

Additional file 3 Table 3. Relative index of inequality (95% CI) for the association of indices of life course socioeconomic position with heavy weekly, heavy daily and problem drinking in men. Click here for file

Additional file 4 Table 4. Relative index of inequality (95% CI) for the association of accumulative indices of life course socioeconomic position with heavy weekly, heavy daily and problem drinking in men. Click here for file
