# Supplementary material for: Problem drinking and exceeding guidelines for 'sensible' alcohol consumption in Scottish men: associations with life course socioeconomic disadvantage in a population-based cohort study
Source: BMC Public Health. 2008 Sep 1;8:302. doi: 10.1186/1471-2458-8-302 (PMC2538536; doi:10.1186/1471-2458-8-302)
Supplement: Additional file 1 — Table 1. Odds ratios (95% CI) for the association of indices of early life socioeconomic position with heavy weekly, heavy daily and problem drinking in men. [file 1471-2458-8-302-S1.doc]

**Table 1. Odds ratios (95% CI) for the association of indices of early life socioeconomic**

**position with heavy weekly, heavy daily and problem drinking in men**

|  |  | **Heavy weekly drinking** | | **Heavy daily drinking** | | **Problem drinking** | |
| --- | --- | --- | --- | --- | --- | --- | --- |
|  |  | **N_cases_/N_risk_** | **Odds ratio (CI)** | **N_cases_/N_risk_** | **Odds ratio (CI)** | **N_cases_/N_risk_** | **Odds ratio (CI)** |
|  |  |  |  |  |  |  |  |
| Father’s social class | Non-manual | 14/85 | 1 (ref) | 34/85 | 1 | 8/85 | 1 |
|  | Skilled manual | 50/268 | 1.16 (0.61, 2.23) | 120/268 | 1.22 (0.74, 2.00) | 41/268 | 1.74 (0.78, 3.87) |
|  | Part-/unskilled | 43/168 | 1.75 (0.89, 3.41) | 76/168 | 1.24 (0.73, 2.11) | 30/169 | 2.08 (0.91, 4.76) |
| P-value (trend) |  |  | 0.057 |  | 0.486 |  | 0.096 |
| Both parents present at 15 yr. | Yes | 91/456 | 1 (ref) | 201/456 | 1 | 69/457 | 1 |
|  | No | 28/111 | 1.35 (0.83, 2.20) | 54/111 | 1.20 (0.79, 1.82) | 17/112 | 1.01 (0.57, 1.79) |
| P-value (difference) |  |  | 0.223 |  | 0.386 |  | 0.983 |
| Number of siblings | 0-1 | 31/136 | 1 (ref) | 57/136 | 1 | 16/137 | 1 |
|  | 2-3 | 31/196 | 0.64 (0.37, 1.11) | 83/196 | 1.02 (0.65, 1.59) | 26/196 | 1.16 (0.60, 2.25) |
|  | 4-5 | 26/125 | 0.89 (0.49, 1.60) | 54/125 | 1.05 (0.65, 1.72) | 20/126 | 1.43 (0.70, 2.89) |
|  | 6+ | 32/118 | 1.26 (0.71, 2.23) | 63/118 | 1.59 (0.97, 2.61) | 24/118 | 1.93 (0.97, 3.84) |
| P-value (trend) |  |  | 0.244 |  | 0.076 |  | 0.043 |
| Education (age left school, yr.) | 15-19 | 27/175 | 1 (ref) | 63/175 | 1 | 17/175 | 1 |
|  | 12-14 | 93/401 | 1.66 (1.03, 2.65) | 195/401 | 1.68 (1.17, 2.43) | 69/403 | 1.92 (1.09, 3.37) |
| P-value (difference) |  |  | 0.036 |  | 0.005 |  | 0.023 |
|  |  |  |  |  |  |  |  |
