# Supplementary material for: Problem drinking and exceeding guidelines for 'sensible' alcohol consumption in Scottish men: associations with life course socioeconomic disadvantage in a population-based cohort study
Source: BMC Public Health. 2008 Sep 1;8:302. doi: 10.1186/1471-2458-8-302 (PMC2538536; doi:10.1186/1471-2458-8-302)
Supplement: Additional file 2 — Table 2. Odds ratios (95% CI) for the association of indices of adult socioeconomic position with heavy weekly, heavy daily and problem drinking in men. [file 1471-2458-8-302-S2.doc]

**Table 2. Odds ratios (95% CI) for the association of indices of adult socioeconomic**

**position with heavy weekly, heavy daily and problem drinking in men**

|  |  | **Heavy weekly drinking** | | **Heavy daily drinking** | | **Problem drinking** | |
| --- | --- | --- | --- | --- | --- | --- | --- |
|  |  | **N_cases_/N_risk_** | **Odds ratio (CI)** | **N_cases_/N_risk_** | **Odds ratio (CI)** | **N_cases_/N_risk_** | **Odds ratio (CI)** |
|  |  |  |  |  |  |  |  |
| Own social class | Non-manual | 33/219 | 1 (ref) | 82/219 | 1 | 18/219 | 1 |
|  | Skilled manual | 59/233 | 1.91 (1.19, 3.07) | 110/233 | 1.49 (1.03, 2.18) | 45/234 | 2.66 (1.49, 4.76) |
|  | Part-/unskilled | 28/124 | 1.64 (0.94, 2.88) | 66/124 | 1.90 (1.22, 2.97) | 23/125 | 2.52 (1.30, 4.88) |
| P-value (trend) |  |  | 0.045 |  | 0.003 |  | 0.004 |
| Employment status | Employed | 70/369 | 1 (ref) | 155/369 | 1 | 48/371 | 1 |
|  | Other | 50/207 | 1.36 (0.90, 2.05) | 103/207 | 1.37 (0.97, 1.93) | 38/207 | 1.51 (0.95, 2.41) |
| P-value (difference) |  |  | 0.142 |  | 0.073 |  | 0.081 |
| Income, GBP/week (quartiles) | 1 (≥207.69) | 31/178 | 1 (ref) | 75/178 | 1 | 22/178 | 1 |
|  | 2 (≥144.50-<207.69) | 31/160 | 1.14 (0.66, 1.98) | 63/160 | 0.89 (0.58, 1.38) | 23/160 | 1.19 (0.64, 2.23) |
|  | 3 (≥90.00-<144.5) | 28/120 | 1.44 (0.81, 2.56) | 54/120 | 1.12 (0.71, 1.79) | 19/122 | 1.31 (0.67, 2.54) |
|  | 4 (<90) | 30/118 | 1.62 (0.92, 2.85) | 66/118 | 1.74 (1.09, 2.79) | 22/118 | 1.63 (0.85, 3.09) |
| P-value (trend) |  |  | 0.067 |  | 0.019 |  | 0.136 |
| Housing tenure | Privately owned | 41/262 | 1 (ref) | 98/262 | 1 | 31/262 | 1 |
|  | Other | 79/314 | 1.81 (1.19, 2.76) | 160/314 | 1.74 (1.25, 2.43) | 55/316 | 1.57 (0.98, 2.52) |
| P-value (difference) |  |  | 0.005 |  | 0.001 |  | 0.062 |
| Household crowding (quartiles) | 1 (<0.33) | 22/108 | 1 (ref) | 43/108 | 1 | 12/109 | 1 |
|  | 2 (≥0.33-<0.43) | 38/172 | 1.11 (0.61, 2.00) | 71/172 | 1.06 (0.65, 1.74) | 26/172 | 1.44 (0.69, 2.99) |
|  | 3 (≥0.43-<0.56) | 34/127 | 1.43 (0.78, 2.63) | 74/127 | 2.11 (1.25, 3.56) | 22/127 | 1.69 (0.80, 3.61) |
|  | 4 (≥0.56) | 26/169 | 0.71 (0.38, 1.33) | 70/169 | 1.07 (0.65, 1.75) | 26/170 | 1.46 (0.70, 3.03) |
| P-value (trend) |  |  | 0.331 |  | 0.396 |  | 0.352 |
| Car ownership | Yes | 55/353 | 1 (ref) | 131/353 | 1 | 39/353 | 1 |
|  | No | 65/223 | 2.23 (1.48, 3.35) | 127/223 | 2.24 (1.59, 3.16) | 47/225 | 2.13 (1.34, 3.38) |
| P-value (difference) |  |  | 0.001 |  | 0.001 |  | 0.001 |
| Marital status | Married | 95/477 | 1 | 211/477 | 1 | 70/478 | 1 |
|  | No longer married | 16/61 | 1.43 (0.77, 2.64) | 28/61 | 1.07 (0.63, 1.83) | 9/62 | 0.99 (0.47, 2.10) |
|  | Single | 9/38 | 1.25 (0.57, 2.73) | 19/38 | 1.26 (0.65, 2.44) | 7/38 | 1.32 (0.56, 3.11) |
| P-value (trend) |  |  | 0.318 |  | 0.488 |  | 0.607 |
|  |  |  |  |  |  |  |  |
