# Supplementary material for: Problem drinking and exceeding guidelines for 'sensible' alcohol consumption in Scottish men: associations with life course socioeconomic disadvantage in a population-based cohort study
Source: BMC Public Health. 2008 Sep 1;8:302. doi: 10.1186/1471-2458-8-302 (PMC2538536; doi:10.1186/1471-2458-8-302)
Supplement: Additional file 3 — Table 3. Relative index of inequality (95% CI) for the association of indices of life course socioeconomic position with heavy weekly, heavy daily and problem drinking in men. [file 1471-2458-8-302-S3.doc]

**Table 3. Relative index of inequality (95% CI) for the association of indices of life course**

**socioeconomic position with heavy weekly, heavy daily and problem drinking in men**

|  | **Heavy weekly drinking** | | **Heavy daily drinking** | | **Problem drinking** | |
| --- | --- | --- | --- | --- | --- | --- |
|  | **N_cases_/N_risk_** | **RII (95% CI)** | **N_cases_/N_risk_** | **RII (95% CI)** | **N_cases_/N_risk_** | **RII (95% CI)** |
|  |  |  |  |  |  |  |
| **Early life** |  |  |  |  |  |  |
| Family structure | 119/567 | 1.83 (0.69, 4.84) | 255/567 | 1.45 (0.63, 3.32) | 86/569 | 1.01 (0.32, 3.21) |
| Father’s social class | 107/521 | 2.29 (1.03, 5.09) | 230/521 | 1.26 (0.66, 2.40) | 79/522 | 2.26 (0.92, 5.57) |
| Education - age left school | 120/576 | 2.67 (1.10, 6.46) | 258/576 | 2.64 (1.32, 5.29) | 86/578 | 2.98 (1.06, 8.34) |
|  |  |  |  |  |  |  |
| **Adult life** |  |  |  |  |  |  |
| Household crowding | 120/576 | 0.80 (0.40, 1.61) | 258/576 | 1.32 (0.74, 2.34) | 86/578 | 1.28 (0.58, 2.85) |
| Marital status | 120/576 | 1.84 (0.68, 5.03) | 258/576 | 1.34 (0.57, 3.17) | 86/578 | 1.34 (0.42, 4.28) |
| Employment status | 120/576 | 1.85 (0.81, 4.21) | 258/576 | 1.87 (0.94, 3.71) | 86/578 | 2.29 (0.90, 5.80) |
| Income | 120/576 | 1.99 (0.99, 4.02) | 258/576 | 1.91 (1.08, 3.39) | 86/578 | 1.89 (0.85, 4.21) |
| Own social class | 120/576 | 2.28 (1.09, 4.78) | 258/576 | 2.45 (1.34, 4.48) | 86/578 | 3.74 (1.58, 8.84) |
| Housing tenure | 120/576 | 3.28 (1.42, 7.60) | 258/576 | 3.02 (1.55, 5.90) | 86/578 | 2.47 (0.96, 6.37) |
| Car ownership | 120/576 | 4.97 (2.20, 11.23) | 258/576 | 5.03 (2.54, 9.96) | 86/578 | 4.52 (1.79, 11.40) |
|  |  |  |  |  |  |  |

Higher scores on each of the socioeconomic variables represent greater disadvantage.
