# Supplementary material for: Problem drinking and exceeding guidelines for 'sensible' alcohol consumption in Scottish men: associations with life course socioeconomic disadvantage in a population-based cohort study
Source: BMC Public Health. 2008 Sep 1;8:302. doi: 10.1186/1471-2458-8-302 (PMC2538536; doi:10.1186/1471-2458-8-302)
Supplement: Additional file 4 — Table 4. Relative index of inequality (95% CI) for the association of accumulative indices of life course socioeconomic position with heavy weekly, heavy daily and problem drinking in men. [file 1471-2458-8-302-S4.doc]

**Table 4. Relative index of inequality (95% CI) for the association of accumulative indices of life course**

**socioeconomic position with heavy weekly, heavy daily and problem drinking in men**

|  | **Heavy weekly drinking** | | **Heavy daily drinking** | | **Problem drinking** | |
| --- | --- | --- | --- | --- | --- | --- |
|  | **N_cases_/N_risk_** | **RII (95% CI)** | **N_cases_/N_risk_** | **RII (95% CI)** | **N_cases_/N_risk_** | **RII (95% CI)** |
|  |  |  |  |  |  |  |
| Accumulated early life score – unadjusted | 106/513 | 2.57 (1.16, 5.70) | 227/513 | 1.70 (0.89, 3.22) | 79/514 | 2.39 (0.98, 5.83) |
| Accumulated early life score – adjusted for later life | 106/513 | 2.01 (0.86, 4.72) | 227/513 | 1.18 (0.59, 2.35) | 79/514 | 1.73 (0.66, 4.51) |
| Accumulated adult life score – unadjusted | 120/576 | 2.26 (1.10, 4.61) | 258/576 | 2.80 (1.56, 5.05) | 86/578 | 3.22 (1.41, 7.35) |
| Life course score – unadjusted | 106/513 | 3.01 (1.40, 6.47) | 227/513 | 2.96 (1.59, 5.52) | 79/514 | 3.36 (1.42, 7.98) |
|  |  |  |  |  |  |  |

Early life characteristics comprise: father’ social class, family structure and education (age left school). Adult life characteristics comprise: employment status, income, housing tenure, household crowding, car ownership, and marital status.

Lifecourse score: all above variables. Again, higher scores on each of the socioeconomic variables represent greater disadvantage.
